# Synthesis of a Novel Electrospun Polycaprolactone Scaffold Functionalized with Ibuprofen for Periodontal Regeneration: An In Vitro andIn Vivo Study

**DOI:** 10.3390/ma11040580

**Published:** 2018-04-10

**Authors:** Fareeha Batool, David-Nicolas Morand, Lionel Thomas, Isaac Maximiliano Bugueno, Javier Aragon, Silvia Irusta, Laetitia Keller, Nadia Benkirane-Jessel, Henri Tenenbaum, Olivier Huck

**Affiliations:** 1 INSERM (French National Institute of Health and Medical Research), UMR 1260, Regenerative Nanomedicine (RNM), FMTS, 67000 Strasbourg, France; s_fareeha_b@hotmail.com (F.B.); davidnicolas.morand@gmail.com (D.-N.M.); isaacmaxi@gmail.com (I.M.B.); lkeller@unistra.fr (L.K.); nadia.jessel@inserm.fr (N.B.-J.); htenen@gmail.com (H.T.); 2 Université de Strasbourg, Faculté de Chirurgie-dentaire, 67000 Strasbourg, France; 3 Institute Pluridisciplinaire Hubert CURIEN (IPHC), Strasbourg 67000, France; lionel.thomas@iphc.cnrs.fr; 4 Department of Chemical Engineering, Nanoscience Institute of Aragon (INA), University of Zaragoza, 50018 Zaragoza, Spain; javier.aragon@unizar.es (J.A.); sirusta@unizar.es (S.I.); 5 Networking Research Center on Bioengineering, Biomaterials and Nanomedicine, CIBER-BBN, 28029 Madrid, Spain; 6 Hopitaux Universitaires de Strasbourg, Pôle de médecine et chirurgie bucco-dentaire, Department of Periodontology, 67000 Strasbourg, France

**Keywords:** regeneration, periodontitis, membrane, GTR, NSAIDs

## Abstract

Ibuprofen (IBU) has been shown to improve periodontal treatment outcomes. The aim of this study was to develop a new anti-inflammatory scaffold by functionalizing an electrospun nanofibrous poly-ε-caprolactone membrane with IBU (IBU-PCL) and to evaluate its impact on periodontal inflammation, wound healing and regeneration in vitro and in vivo. IBU-PCL was synthesized through electrospinning. The effects of IBU-PCL on the proliferation and migration of epithelial cells (EC) and fibroblasts (FB) exposed to *Porphyromonas gingivlais* lipopolysaccharide *(Pg*-LPS) were evaluated through the AlamarBlue test and scratch assay, respectively. Anti-inflammatory and remodeling properties were investigated through Real time qPCR. Finally, the in vivo efficacy of the IBU-PCL membrane was assessed in an experimental periodontitis mouse model through histomorphometric analysis. The results showed that the anti-inflammatory effects of IBU on gingival cells were effectively amplified using the functionalized membrane. IBU-PCL reduced the proliferation and migration of cells challenged by *Pg*-LPS, as well as the expression of fibronectin-1, collagen-IV, integrin α3β1 and laminin-5. In vivo, the membranes significantly improved the clinical attachment and IBU-PCL also reduced inflammation-induced bone destruction. These data showed that the IBU-PCL membrane could efficiently and differentially control inflammatory and migratory gingival cell responses and potentially promote periodontal regeneration.

## 1. Introduction

Periodontal diseases are a group of inflammatory diseases, comprising gingivitis and periodontitis, induced by bacterial infection. Gingivitis is a reversible disease affecting gingival tissues, while periodontitis is irreversible and affects the profound periodontium. Severe periodontitis is the sixth most prevalent disease worldwide affecting around 743 million people [1] and is considered the main cause of tooth loss with an impact on systemic health and quality of life [2]. Periodontitis leads to a progressive destruction of the periodontal tissues including alveolar bone, periodontal ligament and connective tissues. This destructive phenomenon results in periodontal pocket formation defined as the space between pathologically-detached gingiva and tooth surface clinically measured by increased pocket depth (PPD) and decreased clinical attachment level (CAL) [3]. CAL refers to the estimated attachment of tooth-supporting tissues and is directly linked to the prognosis of tooth loss [4].

The main etiological factor of periodontitis is associated with dysbiosis of the periodontal flora resulting in increased proportions of anaerobic bacteria such as *Porphyromonas gingivalis* (*Pg*), a Gram-negative anaerobe often found in severe periodontal lesions, acting through virulence factors such as lipopolysaccharide (*Pg*-LPS) [5]. Periodontal destruction results from the disruption of host-pathogens balance, characterized by sustained inflammation orchestrated by the activation of innate immune response leading to massive recruitment of immune cells, the release of inflammatory mediators including cytokines such as Tumor necrosis factor-alpha (TNF-α) and proteases such as matrix metalloproteinases (MMPs) [6]. 

The aim of periodontal treatment is to reduce bacterial load and suppress inflammation. It consists of oral hygiene instructions, modification of local or systemic risk factors, scaling and root planing (SRP) with, in some clinical scenarios, adjunctive therapeutics such as antimicrobials (antibiotics, antiseptics), probiotics or surgical approaches aiming to reduce bacterial load and sustained tissue inflammation [7,8,9]. The conventional treatment achieves the repair of degraded tissue with some recovery of CAL and reduction in PPD; however, periodontal regeneration still remains elusive [10]. Periodontal regeneration refers to the restoration of destructed tissue to its original state of both form and function [11]. It is of clinical interest to achieve regeneration as it has been associated with long-term benefits including tooth retention, less periodontitis recurrence and less expense for re-intervention [12]. Guided tissue regeneration (GTR) has been considered to be the gold standard for periodontal regeneration for decades and is still considered effective in improving the clinical and radiographic parameters of patients with chronic periodontitis [13,14]. The use of a membrane, as a barrier, prevents early epithelial downgrowth, allowing maturation of bone and periodontal ligament [15]. The use of non-resorbable membranes allows better space maintenance and clinical outcomes; however, it requires a second surgery for removal, increasing the risk of infection. In contrast, the ease of use, gradual degradation and reduced chances of infection render bioresorbable membranes better candidates for GTR [16]. Nevertheless, GTR outcomes could also be impaired by persistent inflammation [17], and the use of new functionalized membranes has been proposed to overcome inflammation and infection-related challenges [18,19,20]. 

The phases during periodontal wound healing are under the control of several growth factors and cytokines. An imbalance between pro- and anti-regenerative molecules can be induced by sustained release of prostaglandins (PGs) and arachidonic acid (AA) metabolites [21]. Therefore, the use of non-steroidal anti-inflammatory drugs (NSAIDs) has been proposed in this regard. NSAIDs block cyclooxygenase (COX), which converts AAs to PGs [22], and previous clinical studies have shown that their use during periodontal treatment leads to PPD reduction and improvement of CAL gain [23], as demonstrated for ibuprofen (IBU) or flurbiprofen. However, their long-term use, especially through systemic delivery, is associated with potential side-effects [24]. 

Currently, multiphasic scaffolds represent one of the newest and very promising nanomaterials in the field of drug delivery, wound healing and tissue engineering. Immediate or modified drug release can be achieved by varying the choice of polymer and the manner of drug loading for nanofiber production [25]. These strategies tend to facilitate the controlled release and local delivery of drugs in a time-dependent manner, rendering it possible to overcome the side-effects of systemic delivery of certain drugs [26]. The functionalization of scaffolds with drugs could be obtained with several methods such as impregnation, incorporation, encapsulation, coating and grafting, hence imparting different advantageous characteristics to the membrane [27]. In this context, a poly-ε-caprolactone (PCL) membrane was evaluated and demonstrated for its pro-regenerative ability in periodontal applications [28]. The PCL membrane has been reported to be biodegradable and biocompatible with enhanced mechanical properties to stabilize the initial clot [29]. Interestingly, functionalization with anti-inflammatory compounds such as alpha-melanocyte-stimulating hormone (α-MSH) has already displayed amplified anti-inflammatory effects associated with anti-soft tissue invasion and anti-fibrotic characteristics [28]. Fascinated by this concept, NSAID-loaded electrospun membranes (ketoprofen/PCL and piroxicam/chitosan) have also been successfully tested for periodontal regeneration in vitro with promising results [30,31,32]. 

The aim of our study was to develop an efficient anti-inflammatory scaffold to overcome the post-operative inflammation after GTR, through localized delivery of IBU from electrospun PCL nanofibers thus, integrating the barrier technique with anti-inflammatory therapy, to assess the biocompatibility and anti-inflammatory properties of the IBU-functionalized PCL membrane (IBU-PCL) and to study its potential pro-regenerative role during periodontal wound healing in vitro and in vivo. The primary expected goal of our synthesized scaffold was to control inflammation and migration of soft tissue-associated cell types and to achieve a short epithelial attachment reinforced by an underlying connective tissue support, thereby eliminating the undesirable long junctional epithelial attachment hindering ad integrum periodontal regeneration.

## 2. Results

### 2.1. Release of IBU from IBU-PCL Membrane

**Figure 1:**
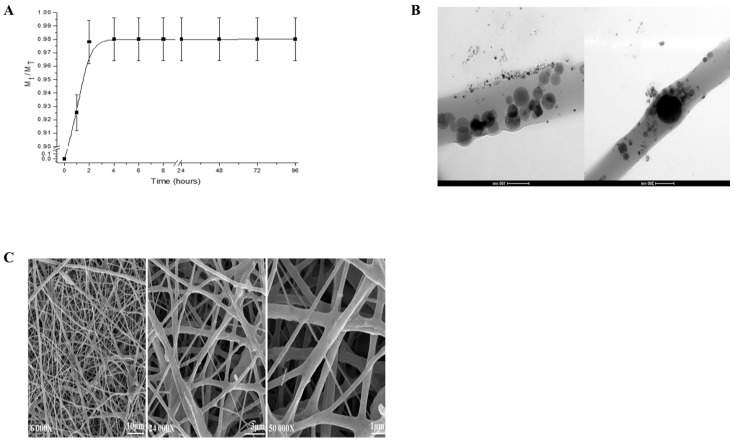
Morphology and analyses (quantitative and qualitative) of IBU-PCL electrospun fibrous scaffolds. In vitro IBU release profile (**A**) and localization of IBU (**B**) within PCL electrospun fibers. 98% of IBU was released from PCL electrospun fibers during the first two hours in PBS. Analysis was determined by UV spectroscopy and transmission electron microscopy observation (TEM). The scale bar of the TEM images represents 100 and 200 nm. The fiber size distribution was obtained by measuring at least 200 fibers in different scanning electron microscopy (SEM) images (**C**) of the IBU-PCL electrospun fibrous membrane. The fiber solution was constituted by 10% of PCL, 20% of non-commercial hydroxyapatite (HAnC), 10% of IBU and 10% of polyvinyl acetate (PVAc) (*w*/*w*), and the fiber diameter was of 374 nm. The scale bar of the SEM images represents 10, 3 and 1 µm.

The release profile showed that IBU exhibited a burst release. The optimal therapeutic concentration of IBU (98%) was achieved after 2 h (Figure 1A). Moreover, it confirmed the encapsulation of the IBU within the PCL phase (Figure 1B). The morphology and fiber diameter distributions of the IBU-PCL membrane exhibited no beads in the fibrous structure, and the fibers were uniform in size and interconnected in order to mimic the natural extracellular matrix (ECM) (Figure 1C). The diameter of fibers was 374 ± 89 nm for the IBU-PCL electrospun fibrous membrane.

### 2.2. IBU-PCL Membrane Reduces Proliferation of Pg-LPS-Stimulated Cells

**Figure 2:**
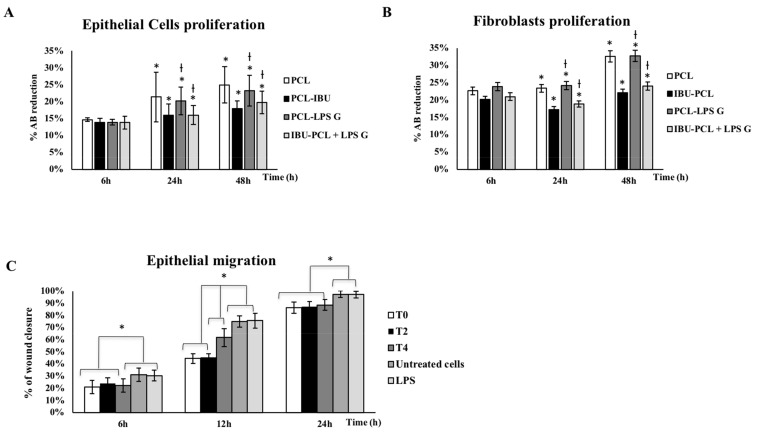
Proliferation of EC (**A**) and FB (**B**) after 6, 24 and 48h and EC migration after 6–24 h (**C**). These different conditions have been measured by using the AlamarBlue test (**A**,**B**). EC and FB proliferation has been evaluated on PCL and IBU-PCL membranes and with or without *Pg*-LPS stimulation. Data are expressed as the mean ± SD. * Difference between cells with or without IBU, *p* < 0.05, † difference between stimulated cells with or without IBU, *p* < 0.05. Epithelial migration has been evaluated through the in vitro scratch assay (**C**) after injection of IBU (50 µg/mL) at baseline (T0), 2 h (T2) and 4 h (T4) at 6, 12 and 24 h in EC stimulated with *Pg*-LPS. Data are expressed as the % of wound closure ± SD; * *p* < 0.05.

To assess if the IBU-PCL membrane influences EC and FB proliferation in an inflammatory context, cells were challenged by *Pg*-LPS during 6–48 h. Exposure to *Pg*-LPS induced an increased proliferation of both EC and FB seeded on the PCL membrane at 24 and 48 h (Figure 2A,B). Interestingly, these increments were not observed for cells seeded on the IBU-PCL membrane. 

To evaluate the impact of IBU on EC migration, a scratch assay has been performed. Data showed that early treatment of *Pg*-LPS-stimulated EC with IBU significantly reduced their migration rate (42% decrease at 12 h; *p* < 0.05) (Figure 2C).

### 2.3. IBU-PCL Membrane Modulates mRNA Expression in Stimulated Cells

**Figure 3:**
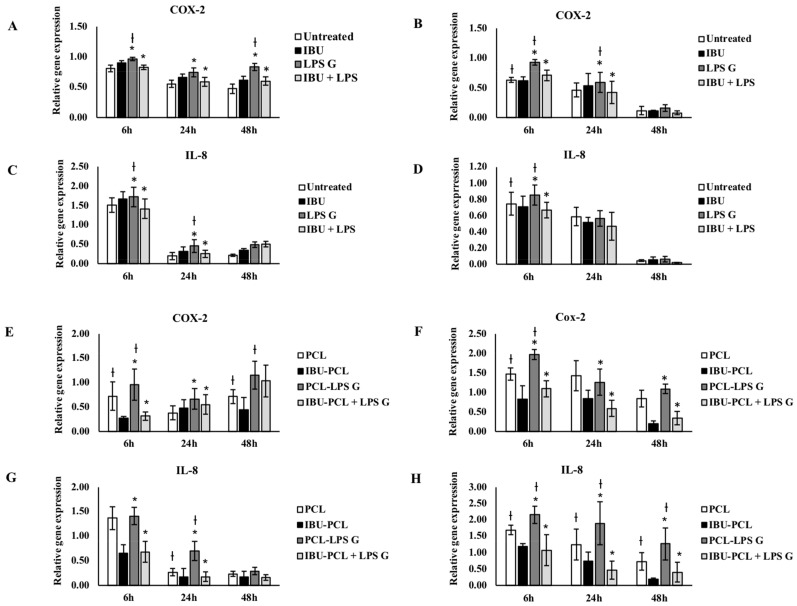
Gene expression of COX-2, IL-8 in EC (**A**,**C**,**E**,**G**) and FB (**B**,**D**,**F**,**H**) cultured on plastic (**A**–**D**) and PCL membrane (**E**–**H**). Relative mRNA levels were analyzed by real-time RT-qPCR for COX-2, IL-8 in EC and FB after 6 h and 24 h. Data are expressed as mean ± SD. † Difference between non-stimulated and stimulated cells, *p* < 0.05; * difference between stimulated cells with or without IBU, *p* < 0.05.

In order to evaluate the anti-inflammatory and pro-regenerative properties of the IBU-PCL membrane, gene expression of COX-2, IL-8 and extracellular matrix (ECM)-related molecules (fibronectin-1, collagen-IV, integrin α3β1 and laminin-5) was measured in cells stimulated by *Pg*-LPS. As expected, exposure to *Pg*-LPS significantly increased gene expression of inflammatory mediators, COX-2 and IL-8 in EC and FB seeded on plastic and on the PCL membrane (Figure 3). Such an increase was counteracted in *Pg*-LPS-stimulated cells seeded on the IBU-PCL membrane emphasizing the anti-inflammatory effect associated with the release or contact between cells and IBU.

**Figure 4:**
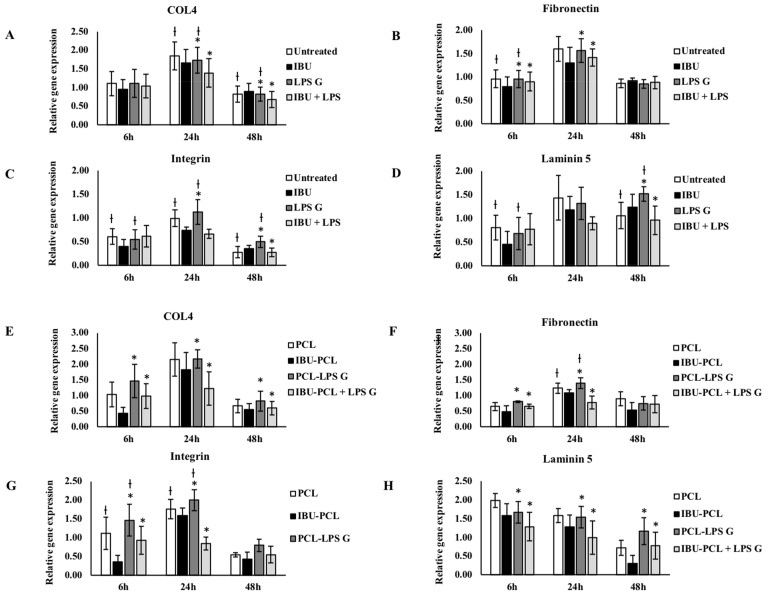
Gene expression of collagen-IV, fibronectin-1, integrin α3β1 and laminin-5 in FB cultured on plastic (**A**–**D**) and PCL membrane (**E**–**H**). Relative mRNA levels were analyzed by real-time RT-qPCR for collagen-IV, fibronectin-1, integrin α3β1 and laminin-5 in FB after 6 and 24 h. Data are expressed as the mean ± SD. † Difference between non-stimulated and stimulated cells, *p* < 0.05; * difference between stimulated cells with or without IBU, *p* < 0.05.

**Figure 5:**
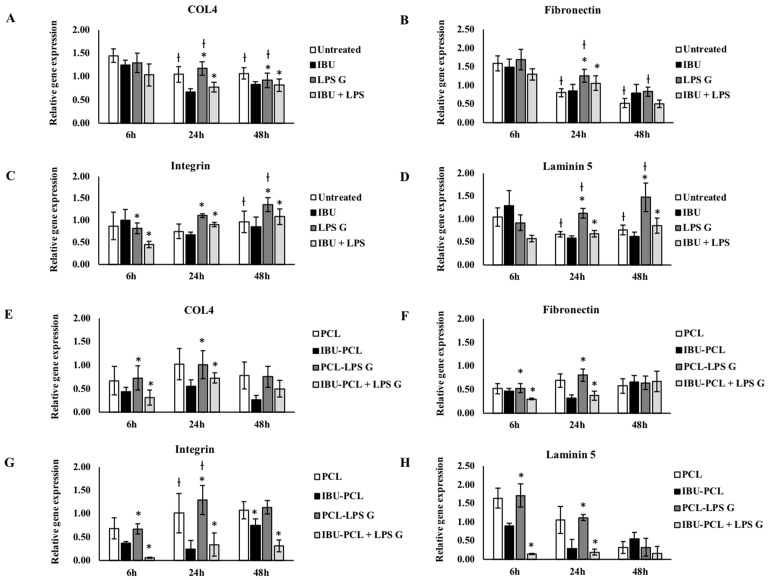
Gene expression of collagen-IV, fibronectin-1, integrin α3β1 and laminin-5 in EC cultured on plastic (**A**–**D**) and PCL membrane (**E**–**H**). Relative mRNA levels were analyzed by real-time RT-qPCR of collagen-IV, fibronectin-1, integrin α3β1 and laminin-5 in EC after 6 and 24 h. Data are expressed as the mean ± SD. † Difference between non-stimulated and stimulated cells, *p* < 0.05; * difference between stimulated cells with or without IBU, *p* < 0.05.

ECM factor expression was also modulated by *Pg*-LPS challenge, and this exposure significantly enhanced fibronectin and laminin-5 expression in FB seeded on plastic and membrane (Figure 4). IBU had no significant effect on mRNA expression of ECM factors in EC and FB not exposed to *Pg*-LPS (Figure 4 and Figure 5) and cultured on plastic compared to their respective controls, whereas, in the presence of *Pg*-LPS, IBU significantly decreased integrin α3β1 expression in EC (Figure 5) and fibronectin-1 expression in FB (Figure 4). In contrast to the cell cultures on plastic, the IBU-PCL membrane decreased mRNA expression of collagen-IV, fibronectin-1, integrin α3β1 and laminin-5 in both non-stimulated and stimulated EC and FB at 6 h. These results showed that embedding of IBU within PCL membrane enhanced its effects on gene expression in a cell-dependent manner. Furthermore, negligible decrease of mRNA expression of COX-2, IL-8 and ECM factors by PCL membrane in cells, both non-stimulated and stimulated with *Pg*-LPS, revealed the non-toxic/non-inflammatory nature of the membrane.

### 2.4. IBU-PCL Membrane Improves Wound Healing in an Induced Periodontitis Mouse Model

**Figure 6:**
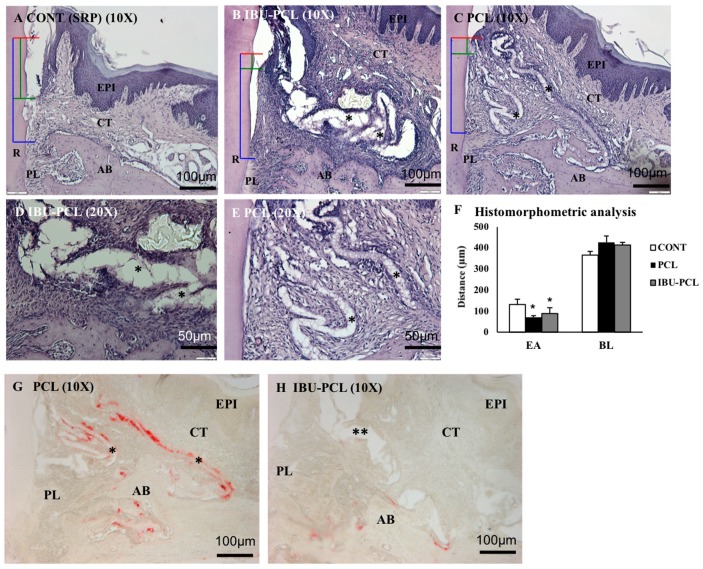
Periodontal wound healing at 22 days. Corresponding histological sections scaling and root planning (SRP) (**A**), IBU-PCL (**B**,**D**), and PCL (**C**,**E**). Red lines = cemento-enamel junction (CEJ); green lines = epithelial attachment level; yellow lines = bone level. PCL and the IBU-PCL membrane are highlighted (*). Histomorphometric analysis (**F**). EA and BL have been measured on histological sections. Distances are expressed as the mean ± SD in µm; * *p* < 0.05. TRAP expression: Few TRAP-positive cells (red staining) were observed on the bone surface at 22 days (**G**,**H**). Numerous TRAP-positive cells were observed around PCL membrane (*), but not around the IBU-PCL membrane (**). EPI: gingival epithelium, CT: gingival connective tissue, AB: alveolar bone, PL: periodontal ligament, R: root, EA: epithelial attachment level, BL: bone loss.

IBU-PCL membrane was surgically placed in an experimental periodontitis mouse model to evaluate its therapeutic potential in vivo (Figure 6). Epithelial attachment (EA) and bone level (BL) were evaluated 22 d after membrane placement. A qualitative improvement of CAL was observed in membrane-treated sites exhibiting a more important connective tissue attachment and a corresponding shorter junctional epithelium in comparison with sites treated with SRP only (*p* < 0.05 for PCL and IBU-PCL vs. control) (Figure 6F). Regarding BL, no improvement was measured in sites treated with either of the membranes in comparison with SRP-treated sites. However, no osteoclastic activity was observed on alveolar bone margins at IBU-PCL-treated sites, while some was detected at PCL-treated sites (Figure 6G,H). Interestingly, some inflammatory cell infiltrate was observed surrounding the membrane (both IBU-PCL and PCL) visibly persistent in the tissue (connective tissue zone) (Figure 6E). In some cases, a space in the fibrous connective tissue organization indicated the presence of a membrane (IBU-PCL) that may have stayed intact for a short duration of time.

## 3. Discussion

Achievement of periodontal regeneration is the ideal goal of periodontal treatment. In this study, an NSAID-loaded scaffold was developed to combine both mechanical properties of a barrier membrane and anti-inflammatory effects of IBU. Herein, we demonstrated the anti-inflammatory and anti-migratory effects of IBU-PCL membrane and its positive effects on periodontal wound healing parameters.

Inflammation is a necessary component of wound healing, which if persists, may hinder tissue regeneration. Excessive inflammation may lead to wound non-closure or development of granulation tissue [32]. Furthermore, activation of COX-2 by bacterial stressors or cytokines (IL-1α, TNF-α) will induce production of PGE2, which has been demonstrated to be involved in the regulation of bone metabolism through activation of related molecular pathways in FB or periodontal ligament cells [33]. Therefore, development of immunomodulatory strategies may be of interest to improve periodontal regeneration outcomes, and several drugs or compounds from synthetic or natural origin have been tested, aiming to reduce inflammatory markers’ levels [20,34,35]. However, systemic delivery may reduce their efficacy and may increase the risk of side-effects. Therefore, new scaffolds based on nanotechnologies were developed to deliver drug to particular tissues or cells [36].

PCL membranes have been previously used to promote periodontal ligament, bone healing [37,38], as a scaffold for periodontal cells [39] and as a drug carrier [40,41]. Biocompatibility of PCL has also been extensively demonstrated with osteoblasts in vitro [42,43] or in vivo [38] and medical-grade PCL is already available [41]. The PCL membrane exhibited a fiber distribution and diameter similar to the ECM combining high infiltration and integration with mechanical properties such as low resorbability and space maintenance [44]. 

Here, IBU was selected and loaded into PCL core-shelled nanofibers, protecting it during the electrospinning process [45]. IBU is a well-described anti-inflammatory drug that has been evaluated in the context of periodontitis [46]. Here, we selected the dose of 50 µg/mL based on the low cytotoxicity and its capability to reduce EC migration. Herein, this dose was able to reduce the expression of inflammatory markers induced by *Pg*-LPS stimulation in both cell types significantly. *Pg*-LPS is a strong inducer of pro-inflammatory responses in gingival EC and FB [28,47]. In this inflammatory model, *Pg*-LPS increased COX-2 and IL-8 expression as previously observed in a cell-dependent manner [48,49,50]. This cell-specific response was also observed in keratinocytes and fibroblasts in skin substitutes [51] and may be explained by the type of Toll-like receptor (TLR) activated [52,53]. In the present study, the IBU-PCL membrane amplified and/or extended over time the anti-inflammatory effect of IBU depending on cell type emphasizing the role of the progressive release by the scaffold as observed for the association with the PLGA membrane [54]. 

The proliferation rate of EC and FB cultured on PCL membranes showed that PCL membranes were biocompatible for gingival cells. Interestingly, the use of PCL membranes delayed cell proliferation, and this effect appeared to be less pronounced in EC than in FB [55]. This reduction of proliferation was amplified with the same dose of IBU in PCL membranes. This difference in proliferation between EC and FB may be due to the surface chemistry and topography, microstructure and mechanical properties of the cultures. Furthermore, PCL membranes functionalized with IBU tend to decrease EC and FB proliferation stimulated by *Pg*-LPS. These results suggest that the use of IBU-PCL membranes may prevent or delay gingival cell migration in an inflammatory context.

Concerning the ECM molecule expressions, IBU downregulated collagen-IV, fibronectin-1, integrin α3β1 and laminin-5 expressions in EC and FB cultured on plastic and PCL membrane. Compared to plastic culture, downregulation of genes was also amplified with the same dose of IBU in PCL. Fibronectin-1 and laminin-5 are essential for periodontal wound healing. Fibronectin-1 constitutes a provisional wound matrix (clot), and laminin-5 is a key ECM component of the intact basement membrane and hemi-desmosomes [56]. Integrin α3b1 is the main molecule by which cells communicate with the ECM mainly through binding to laminin-5 and fibronectin-1 [57]. Collagen-IV constitutes a new matrix that replaces the clot and leads to restoration of both the structure and function of the periodontal basement membrane [58]. These molecules were expressed by keratinocytes, FB [56] and involved in adhesion, migration, proliferation and interaction between the epithelial and connective tissues [54,55,56,57,58]. Furthermore, previous in vivo studies have shown that NSAIDs could significantly inhibit collagen deposition in granulation tissue [59]. Taken together, these data showed anti-inflammatory, anti-proliferative and anti-fibrotic effects of IBU in a time- and cell-dependent manner.

The electrospun PCL nanofibrous scaffold architecturally mimics the ECM in living tissues, but its poor hydrophilicity caused a reduction of its ability of cell adhesion, migration, proliferation and differentiation [60]. However, by combining two or more classes of materials into composites, such as a crystalline ceramic (e.g., HA) and a synthetic polymer (e.g., PCL), scaffolds with improved mechanical properties can be expected [61]. Electrospun composite PCL/nHA (nanohydroxyapatite) nanofibrous membranes improve mineralization of mesenchymal stem cells to promote bone tissue regeneration. nHA is the major inorganic component of the bone matrix, and its specific affinity toward many adhesive proteins and direct involvement in the bone cell differentiation and mineralization processes make nHA especially appealing for applications in the bone regeneration field [62]. nHA has been incorporated in PCL by electrospinning in several studies in vitro [63] and in vivo in a calvarial defect mouse model where association with HA significantly improved bone healing induced by PCL [64]. The HA-coated PCL membrane has favorable effects on proliferation and differentiation of human periodontal ligament cells and might be a candidate material for periodontal tissue regeneration [65]. Similarly, the properties of PCL have also been enhanced by the use of silica [66], and other pretreatments enhancing mineralization would be of interest for bone tissue mineralization and regeneration as demonstrated for cellulose-based porous matrix [67].

Establishment of periodontal destruction in mice is a well-described phenomenon [68], and its use will confer several advantages over the use of large animals in the context of periodontal treatment such as a large number of available kits for analysis, transgenic strains and laboratory considerations (housing, cost). Additionally, it will allow us to investigate the molecular mechanisms regulating the wound healing process or drug application. Infected ligature-induced periodontitis is considered to be a reliable and reproducible model of experimental periodontitis so far as it is site-specific and results, as observed in humans, in rupture and apical migration of the junctional epithelium, inflammatory cell infiltration and time-dependent alveolar bone resorption. In this model, connective tissue and bone loss occur predictably over a period of 7–15 days [69,70]. The ligatures can be inserted and removed on an “as and when required” basis; therefore, using ligatures is a flexible and optimizable method for disease induction [71]. Moreover, the use of *Pg*-infected ligatures supported a long-lasting infection of *Pg* in mice, resulting in alveolar bone breakdown as seen in humans [72]. In our study, we demonstrated, to the best of our knowledge, the feasibility of membrane placement in such an experimental periodontitis model. In vivo, the positive impact of membrane placement on periodontal wound healing and its biocompatibility were observed. However, it is mandatory to understand and control the scaffold degradation process. As tissue ingrowth and maturation are tissue-specific phenomena, a defect filled with immature tissue should not be considered “regenerated”. Hence, many scaffold-based strategies have failed in the past, as the scaffold degradation was more rapid than tissue remodeling or maturation. It is important that the scaffold remains intact as the tissue matures in the scaffold pores, with bulk degradation occurring later [10]. Here, membrane persistence in the connective tissue zone may have hindered bone regeneration, and an optimization of its degradation rate is required. However, the use of bioresorbable PCL membrane at 22 d of periodontal wound healing maintained its primary focus on the soft tissue healing response, whereas a longer time point needs to be evaluated to study the healing response of the bone and periodontal ligament after the resorption of the membrane. As a perspective, combination with specific bone pro-regenerative molecules such as BMP-2 could be performed to improve regeneration of profound periodontium, as it was demonstrated that electrospun PCL functionalized with BMP-2 enhanced bone healing and regeneration [73,74]. Such a combination will be of interest to obtain a better bone healing response, thereby reaching a coordinated soft and hard tissue healing response. 

GTR membranes often suffer exposure to consequent infection and inflammation. The post-operatively persisting inflammation after GTR can worsen the treatment outcomes [17]. Therefore, application of this IBU-loaded anti-inflammatory GTR membrane (IBU-PCL) could be a judicious choice to prevent local post-operative inflammation after further optimization with in vivo and pre-clinical models. In the future, an appropriate combination of antibiotic or growth factors with an anti-inflammatory drug could be ideal to overcome post-operative GTR complications and could be beneficial in striding towards improved periodontal wound healing and regeneration. 

## 4. Materials and Methods

### 4.1. Cell Culture

Human oral epithelial cells (TERT-2 OKF-6, BWH Cell Culture and Microscopy Core, Boston, MA, USA) (EC) were cultivated in Keratinocyte-SFM medium (Life Technologies, Saint-Aubin, France) supplemented with growth supplementation mix and antibiotics (10 U/mL penicillin and 100 µg/mL streptomycin) (Lonza, Levallois-Perret, France). Human oral fibroblasts (FB) were cultivated in RPMI 1640 medium supplemented with 10% fetal bovine serum (Life Technologies, Saint-Aubin, France), 2 mM glutamine, 250 U/mL fungizone and 10 U/mL antibiotics (10 U/mL penicillin and 100 µg/mL streptomycin) at 37 °C in a humidified atmosphere with 5% CO_2_, and the culture medium was changed every 2–3 days as described in Morand et al. [17]. 

### 4.2. Bacterial Culture

The *Pg* strain (ATCC 33277) was purchased from the American Type Culture Collection (ATCC, Manassas, VA, USA). Bacterial culture was performed under strict anaerobic conditions at 37 °C in brain-heart infusion medium supplemented with hemin (5 mg/mL) and menadione (1 mg/mL) purchased from Sigma (St. Louis, MO, USA). For each experiment, bacteria were grown in anaerobic conditions at 37 °C for 4 days, and before use, the bacterial culture was centrifuged, bacteria were washed twice with phosphate buffer saline (PBS) and counted as previously described [51]. Commercial ultrapure *Pg*-LPS was purchased from InvivoGen (San Diego, CA, USA).

### 4.3. Stimulation of Cells with Porphyromonas Gingivalis-Lipopolysaccharide

Twenty-four hours before the experiment, 5.10^4^ EC or 2.10^4^ FB were seeded in each well of a 24-well plate. On the day of the experiment, cells were washed twice with PBS and exposed to *Pg*-LPS stimulation at a concentration of 1 μg/mL. Ibuprofen sodium salt (Sigma, St-Quentin, France) was used as the experimental drug at a concentration of 50 µg/mL.

### 4.4. Electrospinning and Functionalization

IBU-PCL membranes were prepared by electrospinning process using a Yflow 2.2.D-500 electrospinner (Coaxial Electrospinning Machines/R&D Microencapsulation, Malaga, Spain). PCL pellets were dissolved at 10% *w*/*w* (PCL/solvents) in dichloromethyl/dimethyl formamide (DCM/DMF) (1:1), and polyvinyl acetate (PVAc) was dissolved at 10% *w*/*w* (PVAc/solvents) in DMF; these two solutions were stirred overnight at room temperature. To prepare PCL-HAnC-IBU (20% of non-commercial hydroxyapatite (HAnC) and 10% of IBU *w*/*w*) scaffolds, PCL pellets were dissolved in DCM/DMF by stirring overnight at room temperature; then, HAnC and IBU powders were weighed and dispersed with the help of TWEEN^®^ 80 by stirring overnight at room temperature. Both solutions were loaded into 20-mL plastic syringes connected to a coaxial spinneret by plastic tubes having inner needle and outer needle diameters of 0.9 mm and 1.7 mm, respectively, with the outer needle connected to a positive voltage power supply at 13.26 kV. The shell and core flow rates and the spinning distance were fixed at 0.5 mL/h and 19 cm. The spun fibers were collected on a static plate connected to a negative voltage power supply at −2.7 kV. 

### 4.5. Scanning and Transmission Electron Microscopy

In vitro release of IBU was carried out at 37 °C in phosphate buffer saline (PBS) at pH = 7.4. The materials loaded with IBU were immersed in 3 mL of PBS. At predetermined time intervals, aliquots of the dissolution medium were withdrawn, and an equivalent amount of fresh medium was added to maintain a constant dissolution volume. IBU concentration in the aliquots was determined by UV spectroscopy using a Varian Cary 50 PROBE UV-Visible spectrophotometer (Agilent Technologies, Santa Clara, CA, USA) at 221 nm from the standard calibration curve. The prepared fibers were studied by scanning electron microscopy (SEM; CSEM-FEG INSPECT 50, Thermo Fisher Scientific, Waltham, MA, USA) and transmission electron microscopy (TEM; FEI Tecnai F30 and probe aberration-corrected FEI-Titan 60-30, Thermo Fisher Scientific) to characterize fibers’ size and morphology. The size distribution statistics were obtained by measuring at least 200 fibers in different images. Samples for SEM were mounted on metal stubs and sputter-coated with platinum.

### 4.6. Cell Viability Assay

The effect of different doses of IBU on EC and FB viability was analyzed by the AlamarBlue assay (Life Technologies, Saint-Aubin, France). After 6, 24 and 48 h of stimulation, 200 µL of incubation media were transferred to a 96-well plate and measured at 590 and 630 nm in order to determine the percentage of AlamarBlue reduction.

### 4.7. Wound Closure Assay

Cell migration was assessed by the wound-healing “scratch” assay. EC were seeded in 48-well plates at 2.5 × 10^4^ cells/mL and grown until confluence. Cells were washed with PBS. In each well, a scratch was made with the tip of a sterile pipette point (200 µL). Cells were washed again with PBS in order to remove cell debris. In each well, 500 µL of medium containing IBU or only medium were added. The scratch was captured immediately and after 24 h with an optical microscope (Nikon inverted microscope, Eclipse TS100, Nikon, Champigny-sur-Marne, France), and the area of the scratch was calculated with Photoshop CS4. The closure percentage of the scratch was calculated as ((surface of the scratch at time 0 h and surface of the scratch at time 24 h)/(surface of the scratch at time 0 h × 100)), as described in [28]. Only ECs, being the first cell type to migrate to the periodontal wound and hindering tissue regeneration owing to their high proliferation rate, were selected for performing the scratch test.

### 4.8. Real-Time qPCR

To quantify RNA expression, qPCR was performed on the cDNA samples. PCR amplification and analysis were achieved using the CFX Connect™ Real-Time PCR Detection System (Bio-Rad, Miltry-Mory, France). Amplification reactions have been performed using iTaq Universal SYBR Green Supermix (Bio-Rad, Miltry-Mory, France). Beta-actin was used as the endogenous RNA control (housekeeping gene) in the samples. Primer sequences were synthesized by Life Technologies (Saint-Aubin, France). The specificity of the reaction was controlled using melting curve analysis. The expression level was calculated using the comparative Ct method (2^-ΔΔCt^) after normalization to the housekeeping gene. All PCR assays were performed in triplicate, and the results are represented by the mean values. All primers sequences are listed in Supplemental File S1.

### 4.9. Experimental Periodontitis Induction in Mouse Model

To avoid any potential effects of estrogen, only male mice C57BL/6J (n =9) aged 8 weeks were used in this study. All animals were regularly fed and kept in separate cages. All procedures were approved by the local ethics committee and performed according to the regulations for animal experimentation. Mice were examined to evaluate pain and stress, and their weights were monitored daily. Periodontitis was induced in mice by *Pg*-infected ligatures to simulate disease condition comparable to human periodontitis as described previously [68,75]. Briefly, after anesthesia, *Pg*-infected silk ligatures (6-0) were placed repeatedly in the palatal sulcus of the first molar (bilaterally) thrice a week for up to 40 days. The placement of *Pg*-infected ligatures was facilitated by sulcular incisions bilaterally and a drop of a thin mix of Glass Ionomer Cement (Ketac^TM^ Cem radiopaque, 3M ESPE) to retain the ligatures in the sulcus around the cervical areas of the maxillary molars. Infected ligatures were renewed every two days. Gradually, after carrying out a few inductions, the periodontal pocket was well established, and therefore, the ligatures could be retained within the pocket without any need of cement to block them. To ensure uniformity and standardization of the defects, the same procedures were performed bilaterally each time by the same operator to overcome operator bias. After induction, the periodontal lesion was characterized by periodontal pocket formation, soft tissue inflammation associated with bleeding on probing and bone destruction assessed through micro-CT to ensure the uniformity and standardization of defects in terms of the size and morphology before initiating the treatment (average BL = 485 μm) (Supplemental File S2). 

### 4.10. Treatment of Periodontal Defect

**Figure 7:**
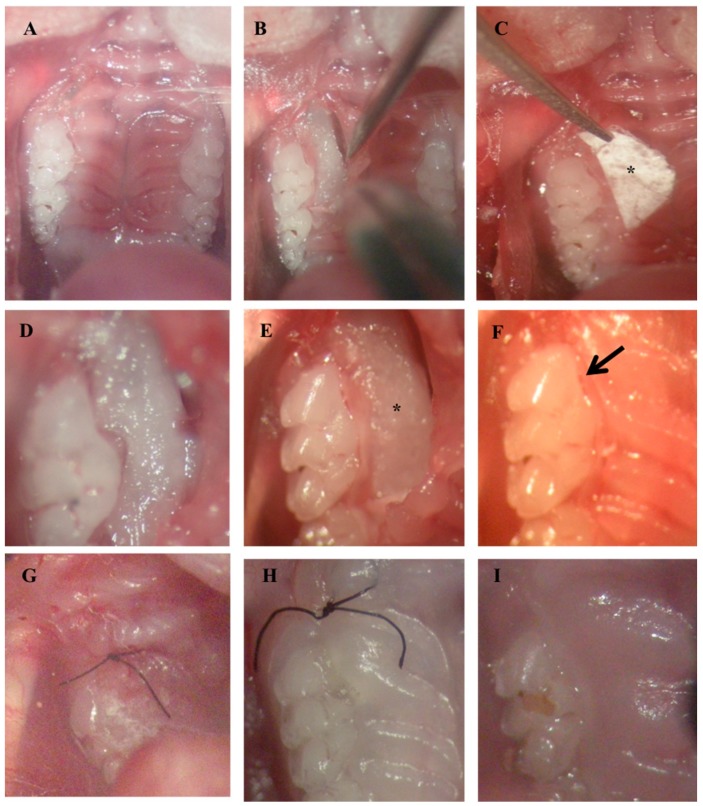
Surgical placement of membrane in the induced periodontitis mice model. Incision (**A**) followed by flap raising for periodontal lesion access and debridement (**B**,**D**). Membrane (*) calibration (**C**) and placement (*) beneath the palatal flap (**E**). Palatal flap covering membrane (arrow) (**F**). Sutures (**G**). Post-surgical views at 7 days (**H**) and 15 days (**I**).

Sulcular incisions were performed bilaterally, along the cervical margins of the maxillary 1st and 2nd molars and extended a little anteriorly on the mesial aspect of the 1st molar to raise the flap efficiently to gain surgical access (Figure 7A,B). SRP was performed at all sites, and PCL or IBU-PCL membranes were surgically placed along the right molars (test sides) in mice (n = 4 and n = 5, respectively). Left molars were treated only by SRP and constituted control sides. Membranes were punched with a 3 mm-diameter cutter. The cut circular pieces of membrane were further cut into two halves. The cut membrane was then placed over the bony defect under the raised flap in such a way that the concave part of the membrane faced and covered the necks of the crowns of the teeth, entering the interdental area, as well, and the rest of the bulk of the membrane was placed flat beneath the flap with its convex side facing towards the palatal midline (Figure 7C–F). The membrane was then sutured to ensure its retention under the flap (Figure 7G). Post-operative wound healing was assessed at 7 and 15 days (Figure 7 H,I). Mice were euthanized with an intraperitoneal lethal injection of pentobarbital (100 mg/kg) (Centravet) 22 days after the treatment.

### 4.11. Tissue Preparation

Tissue fixation was performed by intra-cardiac perfusion with a solution containing 4% paraformaldehyde (PFA) in PBS (pH 7.4). Afterwards, maxillae were dissected and post-fixed by immersion in the same fixative solution overnight at 4 °C. After rinsing with PBS for 24 h, the specimens were demineralized at 4 °C in 15% EDTA at pH 7.4 for 3 weeks with a regular change of solution every 2 days. After extensive washing in PBS, the samples were dehydrated in increasing concentrations of ethanol and toluene before finally embedding in paraffin (Paraplast plus, Sigma). Seven micrometer-thick serial frontal paraffin sections of the maxilla were cut with a microtome.

### 4.12. Histomorphometric Analysis

For histomorphometric evaluation, prepared sections were deparaffinized, rehydrated and stained with hematoxylin. After dehydration, slides were mounted with Distrene-plasticizer-xylene (DPX) resin (Sigma), and computerized images were captured on a microscope (RM 2145 DMRB microscope, Leica, Rueil-Malmaison, France). Palatal root areas of the first molars were analyzed to examine the extension of epithelial downgrowth, connective tissue attachment and alveolar bone loss. For histomorphometric analysis: epithelial attachment (EA) was measured from the cemento-enamel junction (CEJ) to the apical limit of the epithelium, and the alveolar bone level (BL) was measured from CEJ to alveolar bone crest (ABC) using imaging software (ImageJ, 1.46r, National Institute of Mental health (NIMH), Bethesda, Maryland, USA). 

### 4.13. Tartrate-Resistant Acid Phosphatase Activity Assay (TRAP)

Paraffin frontal sections were rehydrated, placed in a fixative solution for 5 minutes and rinsed with water before staining with acetate buffer (at pH 5.2) containing 2.5 mM naphthol AS-TR-phosphate, 0.36 M *N*,*N*-dimethylformamide, 0.1 M sodium tartrate and 4 mM 1,5-naphthalenedisulfonate salt. After staining, sections were rinsed with water and mounted with mounting medium. Using imaging software, TRAP-positive cells were analyzed on the ABC surface at the palatal root and the mesial and distal furcation aspects of the first molar using standardized views. 

### 4.14. Statistical Analysis

All experiments were repeated at least three times (technical and biological replicates), and statistical analysis was performed using the Mann–Whitney test (XLSTAT, Addinsoft France, Paris, France). A probability of a *p*-value < 0.05 was considered significant.

## 5. Conclusions

We developed an efficient anti-inflammatory GTR membrane. Hence, electrospun biodegradable IBU-PCL nanofiber membranes could be an optimal choice for the local prevention of post-surgical inflammation and improved wound healing. Besides, this scaffold may also be used for localized drug delivery of bioactive molecules such as antimicrobials or growth factors, in a dose- and spatially-controlled manner.

## Supplementary Materials

The following are available online at http://www.mdpi.com/1996-1944/11/4/580/s1, File S1: Primers’ sequences, File S2: In vivo micro-computed tomography (micro-CT) analyses.
