# Supplementary material for: Synthesis of a Novel Electrospun Polycaprolactone Scaffold Functionalized with Ibuprofen for Periodontal Regeneration: An In Vitro andIn Vivo Study
Source: Materials (Basel). 2018 Apr 10;11(4):580. doi: 10.3390/ma11040580 (PMC5951464; doi:10.3390/ma11040580)
Supplement: Supplementary file 1 [file materials-11-00580-s001.pdf]

Gene	Primer sequence
β-actin	3’-GATGAGATTGGCATGGCTTT-5’
	5’-CACCTTCACCGTTCCAGTTT-3’
IL-8	3’-GACATACTCCAAACCTTTCCACC-5’
	5’-AACTTCTCCACAACCCTCTGC-3’
COX-2	3’-CCCTTGGGTGTCAAAGGTAA-5’
	5’-GCCCTCGCTTATGATCTGTC-3’
COL-IV	3’-GGATCGGCTACTCTTTTGTGATG-5’
	5’-AAGCGTTTGCGTAGTAATTGCA-3’
Fibronectin-1	3’-GGAGTTGATTATACCATCACTG-5’
	5’-TTTCTGTTTGATCTGGACCT-3’
Integrin α3β1	3’-ACTGTGAAGGCACGAGTGTG-5’
	5’-TGCTGGTTCGGAGGAATAG-3’
Laminin-5	3’-TGACCTTTTCTGGCTCGTCT-5’
	5’-GTTCAGCACAAAGGGCTCTC-3’

Supplemental File S1: Primers sequences

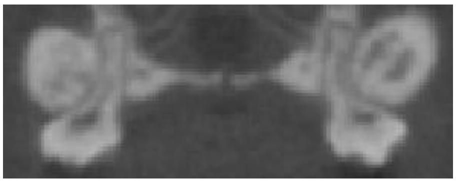

Supplemental File S2: In-vivo micro-computed tomography (micro-CT) analyses

To assess the amount of bone loss following periodontitis induction, bone volume was measured using micro-CT. Acquisitions were performed under general anesthesia after 40 days of induction. A spatial isotropic resolution of 50 μm was used for the acquisitions. Analysis confirmed the presence of periodontal destruction following periodontitis induction.
